# Energy‐Controllable Manipulation on Surface Waves and Propagating Waves by Bifunctional Metasurfaces

**DOI:** 10.1002/nap2.70005

**Published:** 2026-01-13

**Authors:** Shiqing Li, Min Kang, Weikang Pan, Yingying Wang, Yizhen Chen, Xing Peng, Xiangyu Jin, Jianru Li, Shaohua Dong, Lei Zhou, Shulin Sun

**Affiliations:** ^1^ Department of Applied Physics Zhejiang University of Technology Hangzhou China; ^2^ Department of Optical Science and Engineering, Shanghai Engineering Research Centre of Ultra Precision Optical Manufacturing College of Future Information Technology Fudan University Shanghai China; ^3^ Shanghai Key Laboratory of Metasurfaces for Light Manipulation Fudan University Shanghai China; ^4^ College of Intelligent Science and Technology National University of Defense Technology Changsha Hunan China; ^5^ College of Photonics and Optical Engineering Aerospace Information Technology University Jinan China; ^6^ Shandong Key Laboratory of Intelligent Photonic Transmission and Sensing Jinan China; ^7^ Department of Physics State Key Laboratory of Surface Physics and Key Laboratory of Micro and Nano Photonic Structures (Ministry of Education) Fudan University Shanghai China

**Keywords:** energy controllable, far‐field, metasurface, near‐field, surface wave

## Abstract

Manipulating propagating waves (PWs) and surface waves (SWs) in desired manners is important in photonics, but controlling these two electromagnetic modes usually requires separate devices, which is unfavorable for integration optics applications. Recently, although metasurfaces capable of controlling both PWs and SWs have been proposed, they typically rely on dynamically varying the helicities of incident circularly polarized (CP) light, causing complexities in practical applications. In this work, we propose an alternative scheme for designing metasurfaces encoded with both resonance and geometric phases that can simultaneously control PWs and SWs through the co‐ and cross‐polarized output channels under the excitation of a CP wave with a particular helicity. We experimentally prove this concept by realizing two microwave metadevices that can convert normally incident beams with left circular polarization (LCP) into PWs and SWs with predetermined wavefronts. Additionally, we numerically demonstrate how to design metadevices with predetermined energy distributions within these two functional output channels. Our work paves the road to tailor both far‐ and near‐field electromagnetic waves using a single ultra‐compact platform, which can find many applications in integrated optics.

## Graphical abstract

This work presents a bifunctional metasurface for the simultaneous control of propagating and surface waves under the excitation of a circularly polarized wave with a particular helicity. It merges resonance and geometric phases to route wavefronts through distinct channels, validated via microwave experiments. The design also enables tailored energy distribution between channels, offering a compact multifunctional platform for integrated photonics.

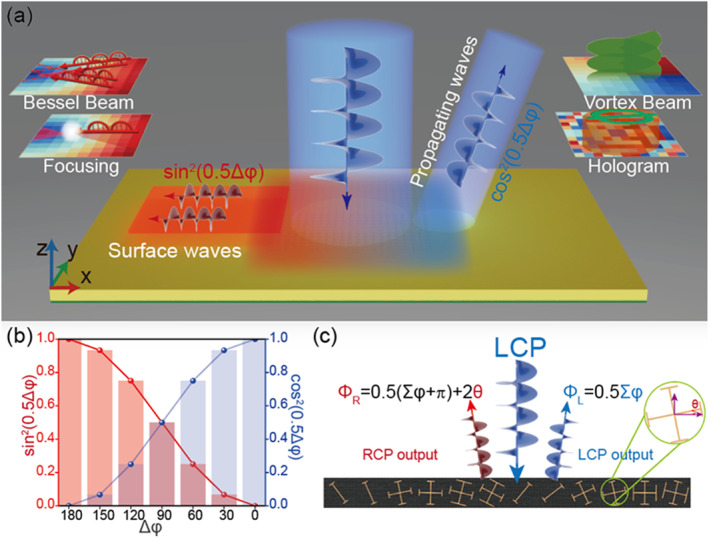

## 1 Introduction

Propagating waves (PWs) and surface waves (SWs)—electromagnetic (EM) eigenmodes bounded at interfaces between metals and dielectrics—are two fundamental modes of EM waves. Manipulating them in desired manners has been a vital goal in photonics [1, 2, 3], which can yield many fascinating applications in information technology, quantum optics, bio/chemical sensing, super‐resolution imaging, and so on. Conventional optical elements (say, lenses) have been proposed to manipulate the wavefronts of PWs or SWs by utilizing the accumulated propagation phases within these devices exhibiting different shapes [4, 5]. However, due to limited choices of electromagnetic parameters in naturally existing materials, these traditional devices inherently suffer from issues of bulky size and restricted functionalities.

Metasurfaces, which can provide abrupt phase shifts at subwavelength scales for incident waves, have demonstrated unprecedented capabilities to manipulate EM waves in the desired manner [6, 7, 8].

Various metadevices have been developed, primarily based on the resonant and Pancharatnam–Berry (PB) phase modulation mechanisms [9, 10, 11, 12, 13]. These devices enable intriguing wavefront manipulation effects for both linearly polarized (LP) and circularly polarized (CP) waves. Examples include anomalous reflection/refraction [14, 15], metaholograms [16, 17, 18], flat lenses [19, 20, 21], complex beam generation [22, 23, 24, 25], and multifunctional metadevices dependent on incident polarization [26, 27, 28, 29, 30, 31, 32] and space [33, 34], among many others [35, 36, 37, 38, 39, 40, 41, 42, 43, 44, 45]. Additionally, metasurfaces have also been proposed to convert PWs into SWs with desired near‐field wavefronts [46, 47, 48, 49, 50, 51, 52]. Despite numerous schemes already proposed for manipulating PWs or SWs, controlling these two different modes usually requires distinct metadevices, which is unfavorable for the integrated optics applications. Recently, a design strategy was proposed to realize metasurfaces encoded with both resonant and PB phases, which can control PWs and SWs independently by changing the helicity of the incident CP wave [51, 52]. However, such bifunctional metasurfaces relying on PB phases inherently work on the cross‐polarization channels, requiring their constituent meta‐atoms to function as ideal half‐wave plates and simultaneously exhibit distinct resonant phases. Otherwise, the co‐polarized normal modes inevitably appear, thereby deteriorating the designed functionalities. Such strict requirements on meta‐atoms make the designs of these bifunctional metadevices complicated.

**FIGURE 1:**
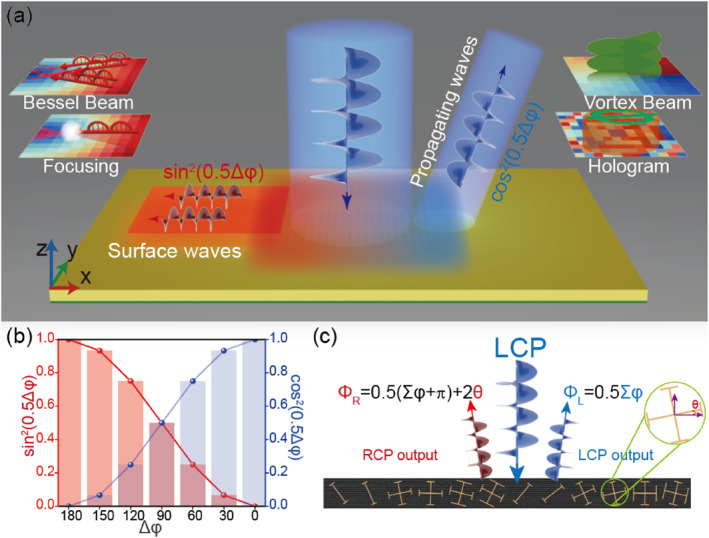
Schematic principle of the proposed metasurfaces. (a) Schematic illustration of the designed metasurface for bifunctional electromagnetic (EM) manipulation of both surface waves (SWs) and propagating waves (PWs) with controllable energy distributions, as illuminated by single‐helicity circularly polarized (CP) waves. The output SW and PW channels enable arbitrary functionalities, such as SW focusing and PW holography, or a SW Bessel beam and a PW vectorial beam. (b) Energy ratio and (c) phase profiles of the co‐ and cross‐polarized output waves, which are determined by the difference of reflection phases $\Delta \varphi =\varphi_{xx}-\varphi_{yy}$ and the sum of reflection phases $\sum \varphi =\varphi_{xx}+\varphi_{yy}$, as well as the orientation θ of the meta‐atoms for two linear polarization cases.

In this work, we present an alternative but novel composite‐phase metadevice design enabling the simultaneous wavefront control of PWs and SWs. Unlike previous studies [51, 52] that rely solely on cross‐polarized channels, our strategy enables the simultaneous control of two distinct EM modes through both co‐ and cross‐polarized field manipulations. This allows the realization of dual functionalities under single‐helicity CP wave illumination, therefore releasing the strict half‐wave plate requirements on constituent meta‐atoms. As a proof of concept, we first design two microwave metadevices and experimentally demonstrate their capabilities to convert incident left circularly polarized (LCP) PWs into both PWs and SWs with predetermined wavefronts through co‐polarized and cross‐polarized output channels, respectively (Figure 1a). Furthermore, we numerically demonstrate that the energy ratio between these two channels can be flexibly controlled by adjusting the phase difference of the meta‐atoms for two orthogonal polarizations, as shown in Figure 1b. Our findings expand the functionalities of metasurfaces, allowing for precise controls of both far‐field and near‐field radiations, and pave the way for diverse practical applications, including ultra‐compact sensing systems, advanced imaging, and logic routing.

## 2 Physical Concept

Let us begin by introducing the proposed mechanism for the simultaneous manipulations of PW and SW. Generally, the reflection properties of the basic meta‐atoms can be characterized by the Jones matrix R(0), in which “0” denotes the orientation angle of the meta‐atoms relative to the *x*‐axis of the laboratory frame. Focusing on the lossless total‐reflection meta‐atoms exhibiting mirror symmetry, we can obtain the following formula: $R\left(0\right)=\left[\begin{array}{cc} r_{uu} & 0\\ 0 & r_{vv} \end{array}\right]=\left[\begin{array}{cc} e^{i\varphi_{uu}} & 0\\ 0 & e^{i\varphi_{vv}} \end{array}\right]$, where *u* and *v* represent the two main axes of the structure, and the reflection amplitude is equal to 1, governed by the energy conservation rule. Supposing that the meta‐atoms are uniformly rotated by a certain angle θ and illuminated by CP waves, the reflection beam would consist of two different components: the spin‐conserved (or co‐polarized) normal mode ${\tilde{r}}_{n}=\frac{1}{2}\left(r_{uu}+r_{vv}\right)$ and the spin‐flipped (or cross‐polarized) abnormal mode ${\tilde{r}}_{a}=\frac{1}{2}\left(r_{uu}-r_{vv}\right)e^{i\sigma \cdot 2\theta }$ (σ=+1 or σ=−1 denoting LCP or right circularly polarized (RCP) state). It should be noted that the abnormal mode will acquire an additional spin‐dependent phase retardation determined by the orientation angle of the meta‐atoms, which is the so‐called PB phase. While these meta‐atoms are excited by LCP light described by |+〉, the reflection properties can be described by the following formula (see Supporting Information S1: Section A for the detailed derivation process):

(1) $$\tilde{R}\left(\theta \right)|+\rangle =\cos\left(0.5\Delta \varphi \right)\cdot e^{i\cdot 0.5\sum \varphi }\cdot |+\rangle +\sin\left(0.5\Delta \varphi \right)\cdot e^{i\cdot 0.5\left(\sum \varphi +\pi \right)}\cdot e^{i\cdot 2\theta }\cdot |-\rangle ,$$

where $\Delta \varphi =\varphi_{uu}-\varphi_{vv}$ and $\sum \varphi =\varphi_{uu}+\varphi_{vv}$ denote the difference and sum of the reflection phases of the meta‐atoms for two orthogonal linear polarization (LP) cases, respectively. Equation (1) implies that there are two output channels with different CPs under the illumination of a single CP light. We purposely design distinct functionalities for these two different modes carrying opposite CP states (|+〉 and |−〉). The phase profile encoded inside the spin‐conserved component, denoted as $\Phi_{1}=0.5\sum \varphi$ (also called the resonant phase), is determined by the sum of the reflection phase for two orthogonal LP illumination cases, which can be adjusted by modifying the geometric parameters of the meta‐atom. Meanwhile, the phase profile of the spin‐flipped mode, represented as $\Phi_{2}=0.5\left(\sum \varphi +\pi \right)+2\theta$, can be viewed as a combination of PB phase and resonant phase. This indicates that the desired functionality for the spin‐flipped abnormal mode can be achieved independently by utilizing an additional degree of freedom, that is, orientation of the meta‐atom. Specifically, the orientation angle associated with the geometric phase can be expressed as $\theta =\frac{1}{2}\left(\Phi_{2}-\Phi_{1}-\frac{\pi }{2}\right)$. The phases encoded inside the two independent channels are totally delinked, as illustrated schematically in Figure 1c.

Additionally, the amplitudes of the spin‐conserved normal mode and the spin‐flipped abnormal mode can also be well controlled. According to Equation (1), the energy ratio between these two output modes, expressed as $\eta =\tan^{2}\left(0.5\Delta \varphi \right)$, is governed by the phase difference Δφ (or the anisotropy) of the meta‐atom, as schematically shown in Figure 1b. Because the resonant phases of the two orthogonal LP components ($\varphi_{uu}$ and $\varphi_{vv}$) are independent, the phase‐modulated wavefronts and the energy weight factor η can be adjusted separately.

While the wavefront engineering based on the PW‐PW conversion process is well‐known, we illustrate how to convert input PW into SW with a desired wavefront. This is another independent functionality achieved by the proposed single device. As discussed in Ref. [51], to realize the PW‐SW conversion, we need to construct a metasurface with a linear retardation phase profile $\Phi \left(x,y\right)=\Phi_{0}+\xi_{x}x$, where the phase gradient $\xi_{x}$ of the metasurface in the *x* direction is larger than the total free‐space wave vector ($\xi_{x}>k_{0}$). Moreover, while the phase profile is further tailored along the *y* direction, the generated SW will exhibit a complex near‐field wavefront. For example, if the phase profile is specified as $\Phi \left(x,y\right)=\Phi_{0}-\xi_{x}x+k_{\text{SW}}\left(\sqrt{y^{2}+F^{2}}-F\right)$ or $\Phi \left(x,y\right)=\Phi_{0}-\xi_{x}x+\xi_{y}\left|y\right|$, the resulting SWs will acquire the wavefront of a focused beam or a Bessel beam.

In short, our strategy involves designing a bifunctional metasurface that can simultaneously tailor the wavefronts of PW and SW through the co‐polarized and cross‐polarized output channels. The implementation of this approach relies on successfully designing a series of meta‐atoms with the phase values of 0.5∑φ that can cover the entire 2π range.

## 3 Meta‐Atom Designs

**FIGURE 2:**
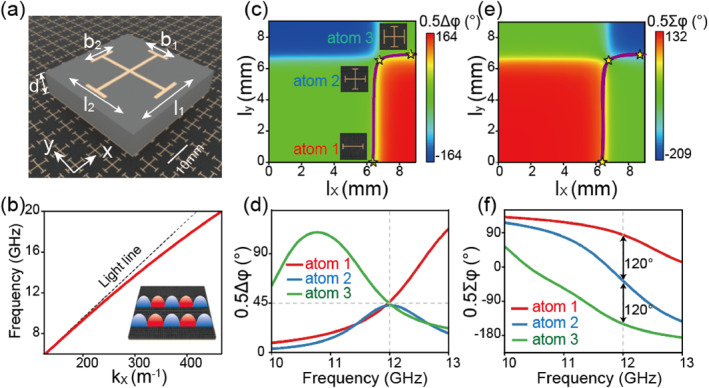
Characterization of the designed meta‐atoms and plasmonic metal. (a) Geometry of the designed meta‐atoms (sized 7 mm × 7 mm) composed of the metallic Jerusalem cross structure array and a flat metal mirror, which are separated by a 1.5‐mm‐thick dielectric spacer. (b) The finite element method (FEM)‐simulated dispersion relation (red line) of the eigen SWs supported by the plasmonic metal, as depicted in the inset. (c, e) Color maps of the simulated (c) 0.5Δφ and (e) 0.5∑φ for the structure depicted in Panel (a) as functions of $l_{x}$ ($l_{x}=l_{1}+b_{1}$) and $l_{y}$ ($l_{y}=l_{2}+b_{2}$) at 12 GHz with the maximum values of 5 mm for $l_{1}$ and $l_{2}$. The purple lines in Panels (c, e) indicate the specific condition of 0.5Δφ=45°. (d, f) Simulated spectra of (d) 0.5Δφ and (f) 0.5∑φ of the three meta‐atoms displayed in Panel (c).

We have selected the microwave regime to demonstrate our scheme. As depicted in Figure 2a, the designed meta‐atoms are a sandwich structure consisting of a metallic Jerusalem cross placed above a ground metallic plane, with a 1.5‐mm‐thick dielectric spacer ($\varepsilon_{r}=3+0.01i$) in between. The presence of the metallic ground plate allows these meta‐atoms to completely reflect input EM waves, with their reflection phases $\varphi_{xx}$ and $\varphi_{yy}$ individually modulated for two orthogonal polarizations. Based on the EM resonances, the reflection phases $\varphi_{xx}$ and $\varphi_{yy}$ are primarily related to the dimensions of the meta‐atom in the *y* direction and *x* direction, respectively (see Supporting Information S1: Section B for more details). Figure 2c,e shows the simulated 0.5Δφ and 0.5∑φ values as functions of $l_{x}$ ($l_{x}=l_{1}+b_{1}$) and $l_{y}$ ($l_{y}=l_{2}+b_{2}$) at the frequency of 12 GHz. The theoretical analysis presented in the previous section indicates that the energy ratio between the LCP and RCP output components is governed by 0.5Δφ, and the phase encoded by the normal mode of the output beam is determined by 0.5∑φ. For example, three representative meta‐atoms with different $l_{x}$ and $l_{y}$ are depicted in Figure 2c, which possess the same value for 0.5Δφ(=π/4) but different values for 0.5∑φ with a constant step of 2π/3. To quantitatively analyze the characteristics of these three meta‐atoms, we have calculated the values of 0.5Δφ and 0.5∑φ as functions of frequency, as plotted in Figure 2d,f. Notably, the three meta‐atoms successfully demonstrate the desired electromagnetic properties at the target frequency of 12 GHz. The conversion efficiency for the cross‐polarized component within the bandwidth can be obtained from Equation (1) (see Supporting Information S1: Section C for the detailed results).

It should be noted that one of the functionalities implemented by the device involves the control of SWs. Considering that natural metals are so conductive that they cannot support surface plasmon modes at microwave frequencies, we have therefore designed an artificial “plasmonic metal” supporting spoof SWs to demonstrate our idea. This design consists of a metallic ground plane covered with a 1.5‐mm‐thick dielectric layer (as shown in the inset of Figure 2b). Figure 2b depicts the FEM‐simulated dispersion relation of the spoof SW mode supported by such a system, which exhibits an eigen wave vector $k_{\text{SW}}=1.035k_{0}$ ($k_{0}$ being the free‐space wave vector) at the target frequency of 12 GHz.

## 4 Metadevices Realizations: Microwave Experiments and Simulations

**FIGURE 3:**
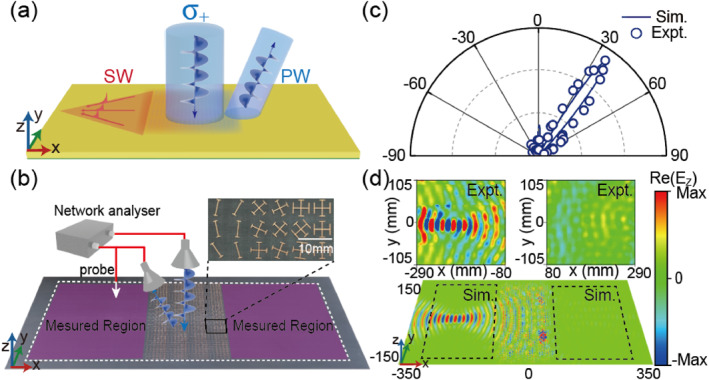
Bifunctional Metadevice I for both PW and SW manipulations. (a) Schematic of the proposed Metadevice I is expected to convert an incident LCP wave to a focused SW in the near field and a deflected PW in the far field, with each channel carrying about half of the incident energy. (b) Schematic of the experimental setup for far‐field and near‐field measurements. The fabricated sample consists of Metadevice I and two connected plasmonic metals. While a source horn antenna emits a CP wave onto Metadevice I, a receiving horn antenna or a monopole probe is utilized to detect the scattered far‐field angular distribution in Panel (c) or the near‐field distribution in Panel (d). All the source antennas and detectors are connected to a network analyzer. (c, d) Scattered far‐field angular distributions in the *xoz* plane and near‐field Re[*E*_*z*_] patterns in the *xoy* plane, as the metadevice is illuminated by an LCP wave at 12 GHz, obtained by microwave experiments and full‐wave simulations.

After developing the physical concept and database of our meta‐atoms, we now demonstrate two bifunctional metadevices for controlling both near‐field and far‐field responses under the illumination of LCP waves. The first metadevice exhibits the following chirality‐dependent phase distributions under LCP wave illumination at the target frequency of 12 GHz:

(2) $$\left\{\begin{array}{c} \varphi^{+}\left(x,y\right)=-\xi_{1}x\\ \varphi^{-}\left(x,y\right)=\varphi_{0}-k_{\text{SW}}\frac{Fx}{\sqrt{y^{2}+F^{2}}}-k_{\text{SW}}\sqrt{y^{2}+F^{2}} \end{array}\right.,$$

where $\xi_{1}=0.595k_{0}$, $k_{SW}=1.035k_{0}$, and *F* = 200 mm. Our analyses, presented in Section 2, indicate that such a metadevice can convert a normally incident LCP beam (*σ* = +1) into two distinct beams, as depicted in Figure 3a. In this case, the co‐polarized beam is anomalously reflected to an oblique direction of $\theta_{r}=\arcsin\left(\xi_{1}/k_{0}\right)=36.5{}^{\circ}$ in the *xoz* plane, whereas the cross‐polarized component is converted into a SW, which is then focused to a point at a distance *F* away from the center of the device. We now employ the meta‐atoms designed in Section 3 to construct this metadevice. To achieve the desired phase gradient $\xi_{1}$ for the co‐polarized output channel, we arrange the three selected meta‐atoms in the following sequence: …, atom 1, atom 1, atom 2, atom 2, atom 3, atom 3, …. According to the relationship between the PB phase and the orientation of the meta‐atoms ($\phi_{\text{PB}}=\sigma 2\theta$), we can unambiguously determine the required orientation angles $\theta_{1}\left(x,y\right)$ of all meta‐atoms located at different positions for the LCP illumination case (σ=+1), that is, $\theta_{1}\left(x,y\right)=\left(\varphi_{0}-k_{\text{SW}}\frac{Fx}{\sqrt{y^{2}+F^{2}}}-k_{\text{SW}}\sqrt{y^{2}+F^{2}}+\xi_{1}x-\frac{\pi }{2}\right)/2$. This strategy guides us to design and fabricate our bifunctional metasurface, as depicted in Figure 3b (see Supporting Information S1: Section D).

We employ FEM simulations and near‐field and far‐field measurements to validate our theoretical predictions. As shown in Figure 3b, one port of the network analyzer is connected to the source horn antenna for emitting normally incident LCP waves onto the sample. The other port is connected either to a receiving horn antenna for measuring the scattered electric field angular distributions of the far‐field PW mode or to a monopole antenna for scanning the pattern of the near‐field SW mode generated on the device. Figure 3c presents the simulated and measured angular distributions of scattered far‐field intensity of the device at the center frequency of 12 GHz. The measured deflection angle of the output LCP PW beam is 36.2°, showing excellent agreement with both theoretical prediction and full‐wave simulation. Additionally, the other functionality, that is, the generation of a focused SW beam, is characterized by the detected Re[*E*_*z*_] field in the *xoy* plane with *z* = 1 mm, as shown in Figure 3d. Here, we employ the near‐field mapping technique to characterize the field pattern of the excited SW. In the measurement process, we illuminate the metasurface with a horn antenna and adopt a monopole probe antenna with a SubMiniature version A connector placed 1 mm above the device, which is carefully aligned along the *z* direction to measure the *E*_*z*_ field component. The probe is driven by a stepper motor to record data at 2 mm intervals. Both the simulated and measured patterns clearly demonstrate that a portion of the incident LCP wave is first converted into SWs, which are then focused to the predefined focal point. The measured focal length is about *F* = 195 mm, agreeing well with both theoretical prediction (*F* = 200 mm) and full‐wave simulation (*F* = 198 mm). FEM simulations indicate that the efficiencies of two functionalities (i.e., PW deflection and SW focusing), defined as the power ratio of the output beam to the input beam, are estimated to be 48.3% and 40.4%, respectively. Although the efficiency of PW deflection is quite close to the ideal efficiency of 50%, the efficiency of the SW focusing beam is below this threshold. This discrepancy may mainly be attributed to the polarization mismatching issue. Specifically, although the incident CP wave comprises both transverse magnetic (TM) and transverse electric (TE) components, only spoof SWs carrying TM polarization are excited on the designed “plasmonic metal,” implying that part of the input energy is lost [50]. Such performance can be further improved by utilizing a more sophisticated “plasmonic metal” in our metadevice, which supports not only TM‐polarized but also TE‐polarized SWs [49].

When the helicity of the incident beam is switched to RCP (σ=−1), the phase profiles of the co‐ and cross‐polarized output beams change to the following forms:

(3) $$\left\{\begin{array}{c} \varphi^{+}\left(x,y\right)=-\xi_{1}x-2\theta_{1}\left(x,y\right)=-\varphi_{0}+k_{\text{SW}}\frac{Fx}{\sqrt{y^{2}+F^{2}}}+k_{\text{SW}}\sqrt{y^{2}+F^{2}}-2\xi_{1}x+\frac{\pi }{2}\\ \varphi^{-}\left(x,y\right)=-\xi_{1}x \end{array}\right..$$

This indicates that the output beams comprise an anomalously reflected PW beam and a defocused SW beam (see simulated results in Supporting Information S1: Section E). According to Equation (3), the simulated scattered‐field angular distributions confirm that the co‐polarized output beam is deflected along the same direction of *θ*_*r*_ = 36.5°. Because this co‐polarized normal mode is determined only by the spin‐independent resonance phase (0.5∑φ), the deflection angles are thus identical for both LCP and RCP illumination. Meanwhile, the metasurface allows for the third functionality, that is, the generation of a defocused SW, in the cross‐polarized component due to the nature of the spin‐dependent PB phase.

**FIGURE 4:**
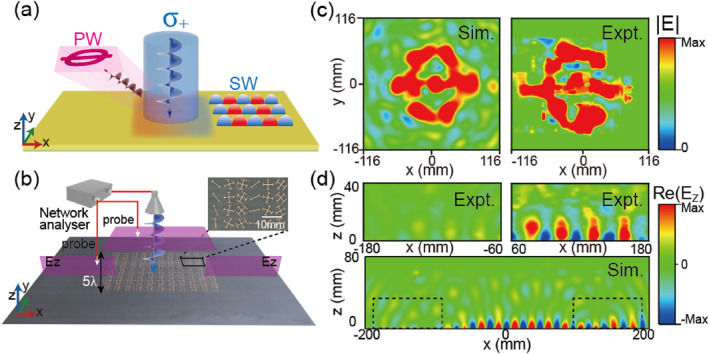
Bifunctional Metadevice II for both PW and SW manipulations. (a) Schematic of the proposed Metadevice II for simultaneously generating holographic images in the far‐field channel and exciting SWs in the near‐field channel. (b) Experimental setup for mapping electric field patterns, which consists of the fabricated Metadevice II connected by two plasmonic metals, a source horn antenna, a monopole detection antenna, and a network analyzer. The right panel depicts an image of part of the fabricated metadevice sample. (c, d) Measured and simulated Re[*E*_*z*_] field patterns of (c) the holographic image projected in the far‐field channel and (d) a unidirectional plane‐wave‐like SW beam along the +*x* direction excited in the near‐field channel, as the metadevice is illuminated by an LCP wave at 12 GHz.

We employ the proposed strategy to further demonstrate, both numerically and experimentally, another metadevice capable of generating a far‐field hologram and a near‐field SW beam (as schematically depicted in Figure 4a). The spin‐dependent phase profiles of this metadevice for the LCP illumination case (σ=1) should satisfy the following equations:

(4) $$\left\{\begin{array}{c} \varphi^{+}\left(x,y\right)=\xi_{2}x\\ \varphi^{-}\left(x,y\right)=\xi_{2}x+\sigma \cdot 2\theta_{2}\left(x,y\right)=\phi_{\text{CGH}} \end{array}\right.,$$

where $\xi_{2}=1.19k_{0}$ is the phase gradient supplied by Metadevice II, and $\phi_{\text{CGH}}$ represents the phase distribution obtained using the Gerchberg–Saxton (GS) algorithm for generating the image of the letter “Φ” in the *xoy* plane (*z* = 125 mm).

We finally design and fabricate the second metadevice (see Supporting Information S1: Section D) based on the phase distributions $\varphi^{+}\left(x,y\right)$ and $\varphi^{-}\left(x,y\right)$ described in Equation (4). We then use the techniques displayed in Figure 4b to characterize the functionality of the device under LCP excitation. Figure 4c presents the simulated and measured electric field patterns in the *xoy* plane at *z* = 125 mm at the center working frequency of 12 GHz, clearly showing an image of “Φ,” as predicted by our theoretical design. The second functionality, that is, the excitation of a plane‐wave‐like SW beam, is characterized by the Re[*E*_*z*_] field pattern in the *xoz* plane (*y* = 0 mm), as shown in Figure 4d. Both numerical simulations and experimental measurements clearly demonstrate that a portion of the incident LCP wave is converted into SW, which is also in good agreement with the theoretical prediction. We can further predict that although the incident beam is changed to an RCP wave, the co‐polarized component will retain the same function of SW excitation. In addition, the holographic image governed by the cross‐polarized component is changed (see Supporting Information S1: Section E). We have numerically demonstrated that the two bifunctional metasurfaces for SW and PW manipulation operate over bandwidths of approximately 1 and 1.5 GHz, respectively (see detailed results in Supporting Information S1: Section F).

**FIGURE 5:**
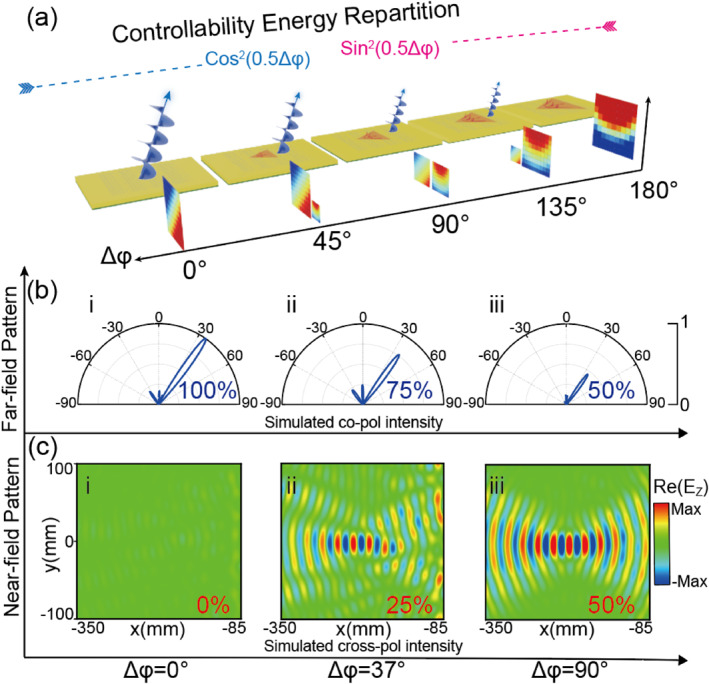
Numerical demonstration of the energy distribution controllability of two channels generated by the bifunctional metasurface. (a) Schematic illustration of controllable energy distribution in the far‐field and near‐field output channels for a series of metadevices with different phase differences. (b, c) Simulated scattered far‐field angular distributions and near‐field focusing patterns of the metadevices with different phase differences (Δ*φ* = 0°, 37°, 90°), illuminated by an LCP beam at 12 GHz.

The proposed theoretical framework enables the flexible control of the energies distributed in the two output channels through tailoring the phase difference Δ*φ* of the metadevice between two orthogonal LPs. As schematically shown in Figure 5a, the evolution of energy distribution across the two output channels with respect to Δ*φ* is numerically demonstrated by a series of metadevices. Building upon the design principles established for Metadevice I, these metadevices are created to simultaneously generate a deflected PW beam in the co‐polarized channel and a focused SW in the cross‐polarized channel under LCP wave illumination. Three metadevices composed of different meta‐atoms with Δ*φ* = 0°, 37°, and 90° are developed to validate the controllable energy distributions between the two functionalities (see Supporting Information S1: Section G), with the simulated results displayed in Figure 5b,c. We can note that the normalized far‐field intensity of the co‐polarized channel decreases as Δφ increases, whereas the electric field intensity of the cross‐polarized near‐field channel exhibits an increasing trend. Specifically, the simulated absolute efficiencies of the co‐polarized and cross‐polarized channels of three metadevices for Δ*φ* = 0°, 37°, and 90° are 87.2% versus 0.01%, 71.6% versus 18.5%, and 48.3% versus 40.4%, respectively. Full‐wave simulations show good agreement with theoretical prediction according to Equation (1), further confirming the energy‐tuning capabilities of the bifunctional metasurface. Moreover, as the phase difference further increases from 90° to 180°, the functionality achieved by the far‐field channel disappears, and the other functionality based on the near‐field channel dominates.

## 5 Conclusions

In summary, we have introduced a novel approach for creating a composite‐phase metasurface that can simultaneously modify the wavefronts of SWs and PWs under the excitation of CP wave with a specific helicity. By integrating two distinct mechanisms (geometric phase and resonance phase), we can independently modulate the phase profiles for the co‐polarized and cross‐polarized channels, allowing for individual wavefront manipulations in near‐field and far‐field channels. As a proof of concept, we have meticulously designed and fabricated two metadevices working at the microwave regime and successfully validated the scheme. Bifunctional wave controls of two devices, including anomalous PW deflection and focused SW generation, and PW hologram and plane‐wave‐like SW excitation, are perfectly demonstrated through full‐wave simulations and experimental measurements. The total efficiencies for these two devices reach the high values of 88.7% (including 48.3% in the far‐field channel and 40.4% in the near‐field channel) and 80.8% (including 37.2% in the far‐field channel and 43.6% in the near‐field channel), according to numerical simulations. Moreover, this method enables the flexible assignment of energy distributions across these two output channels. By optimizing the quality factors of the meta‐atoms or introducing structural resonances at distinct frequencies based on more sophisticated meta‐atoms, we can expand the operational bandwidth [53] or achieve frequency multiplexing [54] for the proposed device. This research represents a meaningful future direction for this field. Our results establish a new platform for controlling both PWs and SWs using a single ultra‐compact metadevice, which could stimulate a variety of practical applications, such as on‐chip photonic devices, sensing, and super‐resolution imaging across different frequency regimes.

## Author Contributions

All authors have accepted responsibility for the entire content of this manuscript and consented to its submission to the journal, reviewed all the results and approved the final version of the manuscript. S.L., S.S., L.Z., and S.D. conceived the idea of this study. M.K., W.P., and Y.W. performed numerical simulations. M.K., Y.W., X.J., and J.L. conducted the measurement and analysis. M.K., Y.C., and X.P. fabricated the sample. S.S., S.D., and L.Z. supervised the project.

## Funding

This work was supported by the National Natural Science Foundation of China (Grants 12104401, 52305594, 12374344, 12221004, and 62192771), the Natural Science Foundation of Zhejiang Province (LZ25A040002), and the National Key Research and Development Program of China (Grants 2024YFB29NL00100, 2022YFA1404700, and 2020YFA0710100).

## Conflicts of Interest

The authors declare no conflicts of interest.

## Supporting information


Supporting Information S1

## Data Availability

The datasets generated during and/or analyzed during the currentstudy are available from the authors upon reasonable request.
