# Supplementary material for: Energy‐Controllable Manipulation on Surface Waves and Propagating Waves by Bifunctional Metasurfaces
Source: Nanophotonics. 2026 Jan 13;15(1):e70005. doi: 10.1002/nap2.70005 (PMC12965005; doi:10.1002/nap2.70005)
Supplement: Supplementary file 1 — Supporting Information S1 [file NAP2-15-e70005-s001.docx]

**Supporting Information: Energy-controllable manipulation on surface waves and propagating waves by bi-functional metasurfaces**

Shiqing Li^1,a^, Min Kang^1,a^, Weikang Pan^2,3,a^, Yingying Wang^2,3^, Yizhen Chen^2,3^, Xing Peng^4^, Xiangyu Jin^2,3^, Jianru Li^2,3^, Shaohua Dong^5,6*^, Lei Zhou^3,7*^ and Shulin Sun^2,*^

^1^Department of Applied Physics, Zhejiang University of Technology, Hangzhou 310023, China

^2^Shanghai Engineering Research Centre of Ultra Precision Optical Manufacturing, Department of Optical Science and Engineering, College of Future Information Technology, Fudan University, Shanghai 200433, China

^3^Shanghai Key Laboratory of Metasurfaces for Light Manipulation, Fudan University, Shanghai 200433, China

^4^College of Intelligent Science and Technology, National University of Defense Technology, Changsha, Hunan 410073, China

^5^College of Photonics and Optical Engineering, Aerospace Information Technology University, Jinan 250299, China

^6^Shandong Key Laboratory of Intelligent Photonic Transmission and Sensing, Jinan 250299, China

^7^State Key Laboratory of Surface Physics and Department of Physics and Key Laboratory of Micro and Nano Photonic Structures (MOE), Fudan University, Shanghai 200433, China

*E-mail: [sls@fudan.edu.cn](mailto:sls@fudan.edu.cn)

*E-mail: [phzhou@fudan.edu.cn](mailto:phzhou@fudan.edu.cn)

*E-mail: [lightdong@yeah.net](mailto:lightdong@yeah.net)

Number of Pages: 9

Number of Figures: 6

References: 2

Contents

[A. Derivation of Jones matrix for the meta-atoms 3](#_Toc210312732)

[B. Reflection phase profiles of the meta-atoms for orthogonal LP cases 4](#_Toc210312733)

[C. Conversion efficiencies of the three meta-atoms 5](#_Toc210312732)

[D. Structural details of two fabricated meta-devices in Fig. 3 and Fig.4 6](#_Toc210312734)

[E. The third functionalities of metasurface I and II under RCP illumination 8](#_Toc210312735)

[F. Bandwidth of the meta-devices for bifunctional SW and PW manipulations 9](#_Toc210312734)

[G. Structural details of three bi-functional metadevices in Fig. 5 11](#_Toc210312736)

[References: 12](#_Toc210312737)

# A. Derivation of Jones matrix for the meta-atoms

The Jones matrix of the general reflective meta-atoms arranged in periodic array can be expressed as follows:, where is the phase difference between two orthogonal linear polarizations (LP), i.e., . When the meta-atoms are rotated by an angle of *θ*, their Jones matrix can be described in a new form:

(S1).

Here, $M(\theta)$ represents a rotation operation. For LCP wave illumination case, the reflected *E*-fields of the meta-atoms can be written as follow:

(S2).

Here, $\Sigma\phi=\phi_{xx}+\phi_{yy}$ is the sum of the reflection phases for two orthogonal LP cases. And $\frac{1}{2}\sum\phi$ is provided by resonance phases which can be modulated by varying the dimensions of meta-atoms. Additionally, $2\theta\left(x,y\right)$is the geometric phase obtained by the cross-polarized mode, which is an additional degree of freedom via changing the orientation angle of the meta-atoms. For RCP illumination case, the geometric phase becomes $-2\theta\left(x,y\right)$ due to the spin-dependent nature of geometric phase [1,2].

# B. Reflection phase profiles of the meta-atoms for orthogonal LP cases


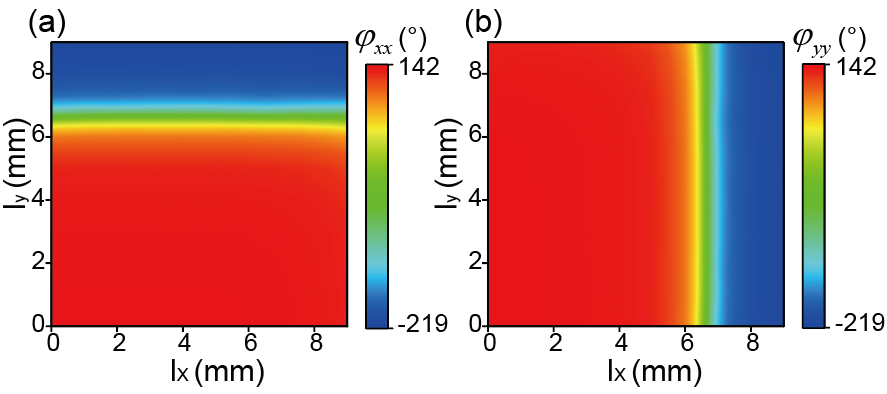


Figure S1. Pseudocolor maps of simulated co-polarization reflection phase profiles of the meta-atom with different values of $l_{x}(l_{x}=l_{1}+b_{1})$and $l_{y}(l_{y}=l_{2}+b_{2})$ under (a) x-linearly polarized and (b) y-linearly polarized waves at 12 GHz.

# C. Conversion efficiencies of the three meta-atoms.


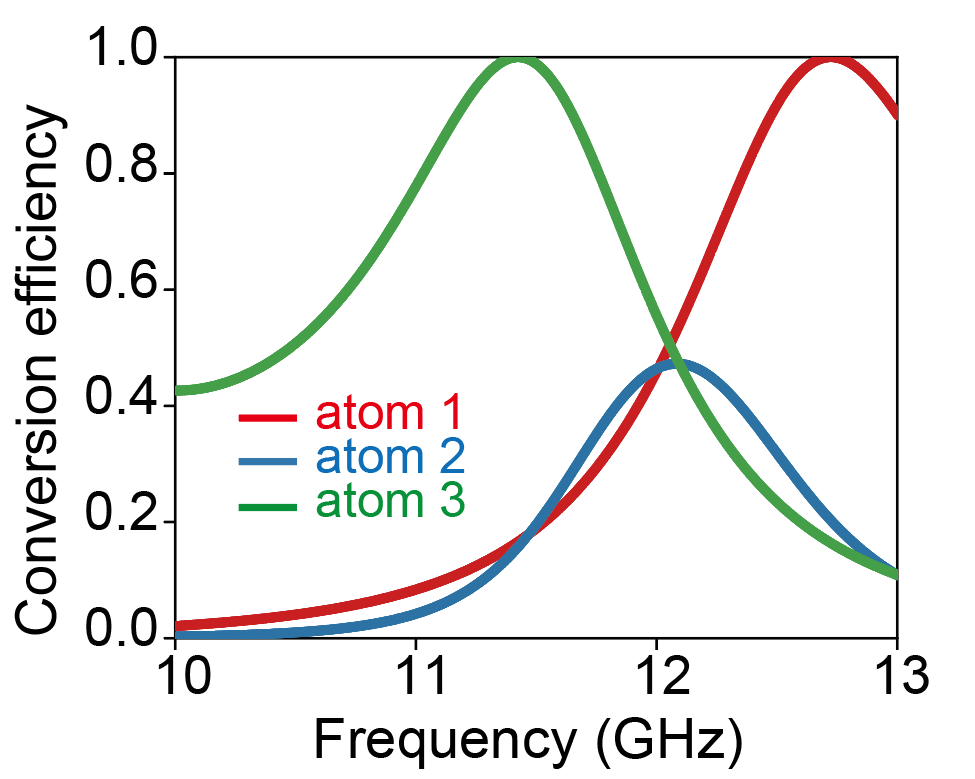


Figure S2. Conversion efficiencies of the three meta-atoms.

# D. Structural details of two fabricated meta-devices in Fig. 3 and Fig.4


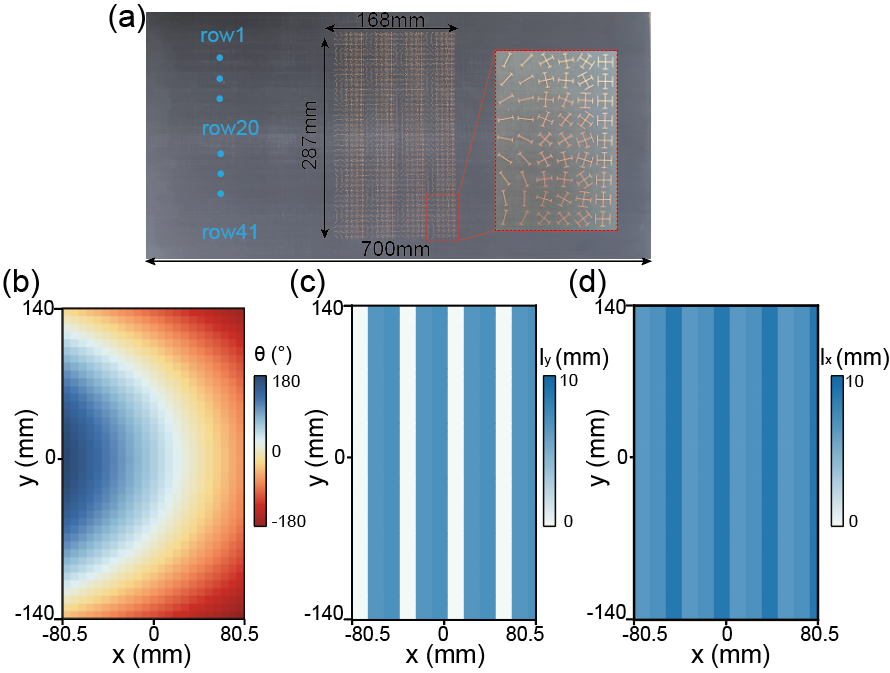


Figure S3. (a) Top-view picture of the fabricated meta-device I composed of 24$\times$41 meta-atoms with different geometries and orientations and the plasmonic metal. The bottom-right region of the meta-device I is shown in the inset. (b-d) Distributions of (b)$\theta(x,y)$, (c) $l_{y}(x,y)$ and (d) $l_{x}(x,y)$for the designed/fabricated meta-device I for bi-functional PW and SW manipulations.


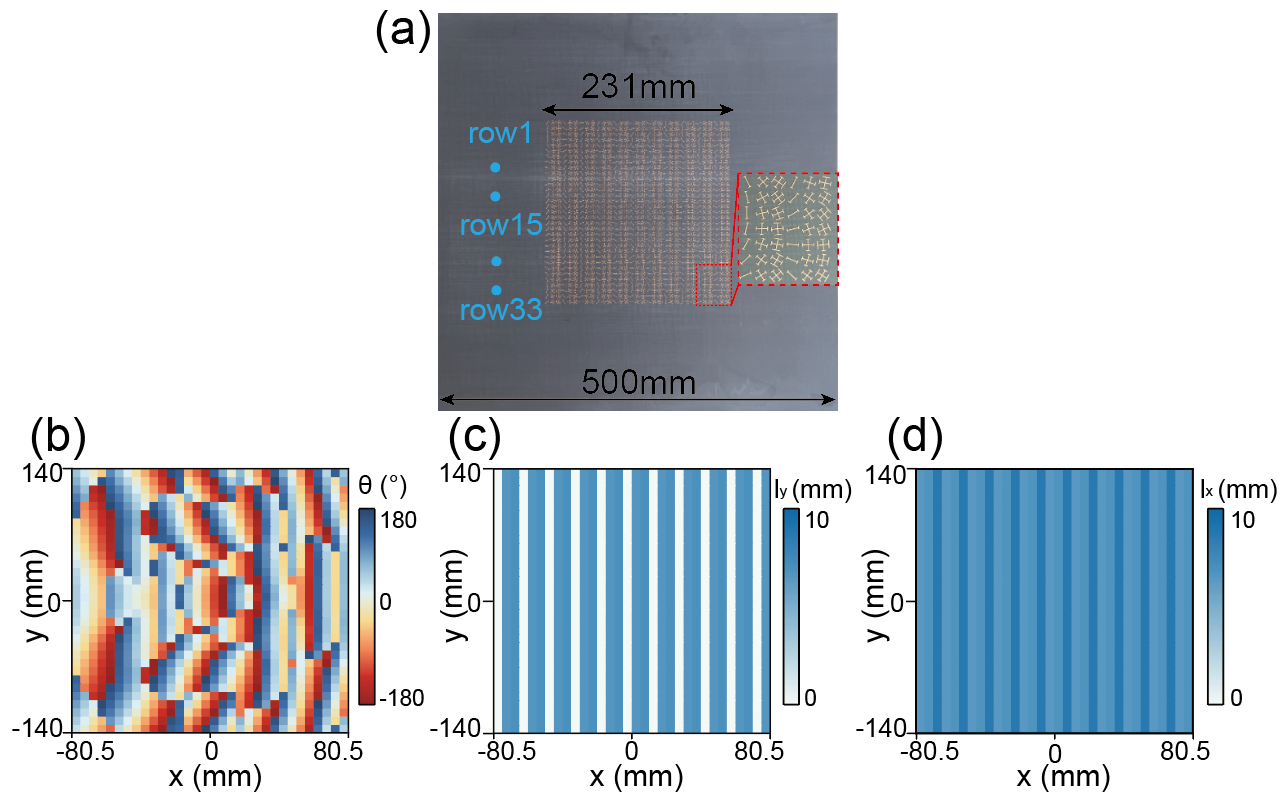


Figure S4. (a) Top-view picture of the fabricated meta-device II composed of 33$\times$33 meta-atoms with different geometries and orientations and the plasmonic metal. The bottom-right region of the meta-device II is shown in the inset. (b-d) Distributions of (b)$\theta(x,y)$, (c) $l_{y}(x,y)$ and (d) $l_{x}(x,y)$for the designed/fabricated meta-device II for bi-functional PW and SW manipulations.

# E. The third functionalities of metasurface I and II under RCP illumination


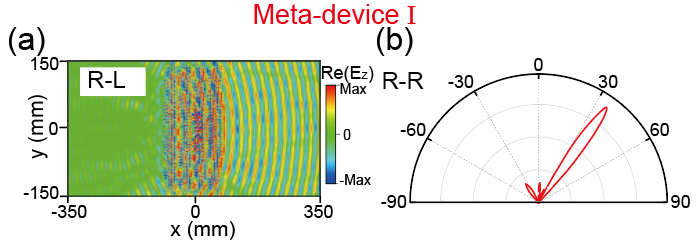


Figure S5. Simulated (a) near-field Re[*E_z_*] pattern in the *xoy* plane 1 mm above the plasmonic metal of cross-polarized mode and (b) scattered far-field angular distribution of co-polarized mode generated by meta-device I depicted in Fig. 3 under the illumination of RCP wave at 12 GHz.


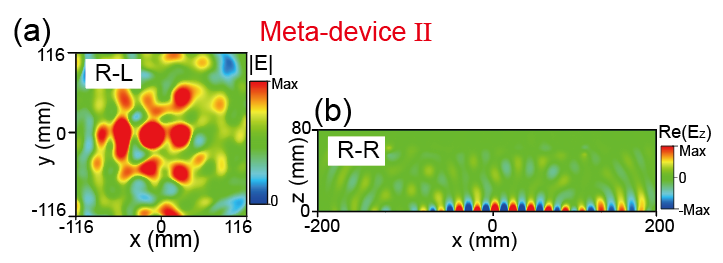


Figure S6. Simulated (a) electric-field patterns in the *x-y* plane at *z* = 125 mm of cross-polarized mode and (b) near-field Re[*E_z_*] pattern of the co-polarized mode of meta-device II shown in Fig. 4 under the illumination of RCP wave at 12 GHz.

# F. Bandwidth of the meta-devices for bifunctional SW and PW manipulations


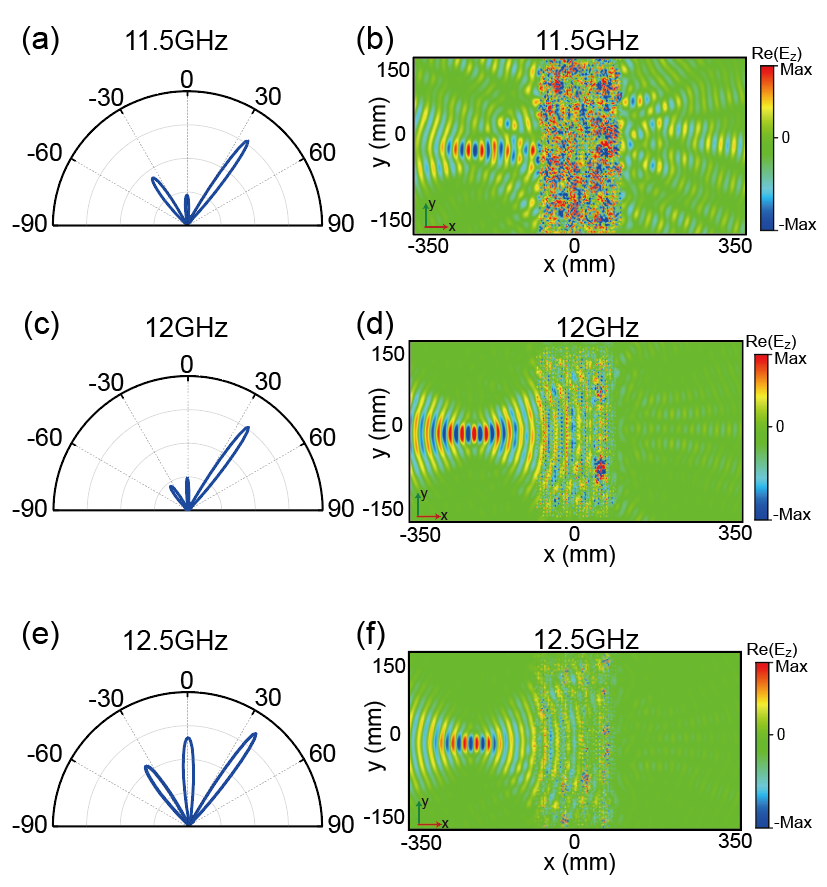


FIG. S7. Simulated (a,c,e) scattered far-field angular distributions of co-polarized mode and (b,d,f) near-field Re[*E_z_*] patterns of cross-polarized mode on the *y*-*z* plane at x=0, generated by meta-device I under LCP wave illumination from 11.5 to 12.5 GHz. It is noted that the working band of such bifunctional metasurface is about 1 GHz (11.5- 12.5 GHz).


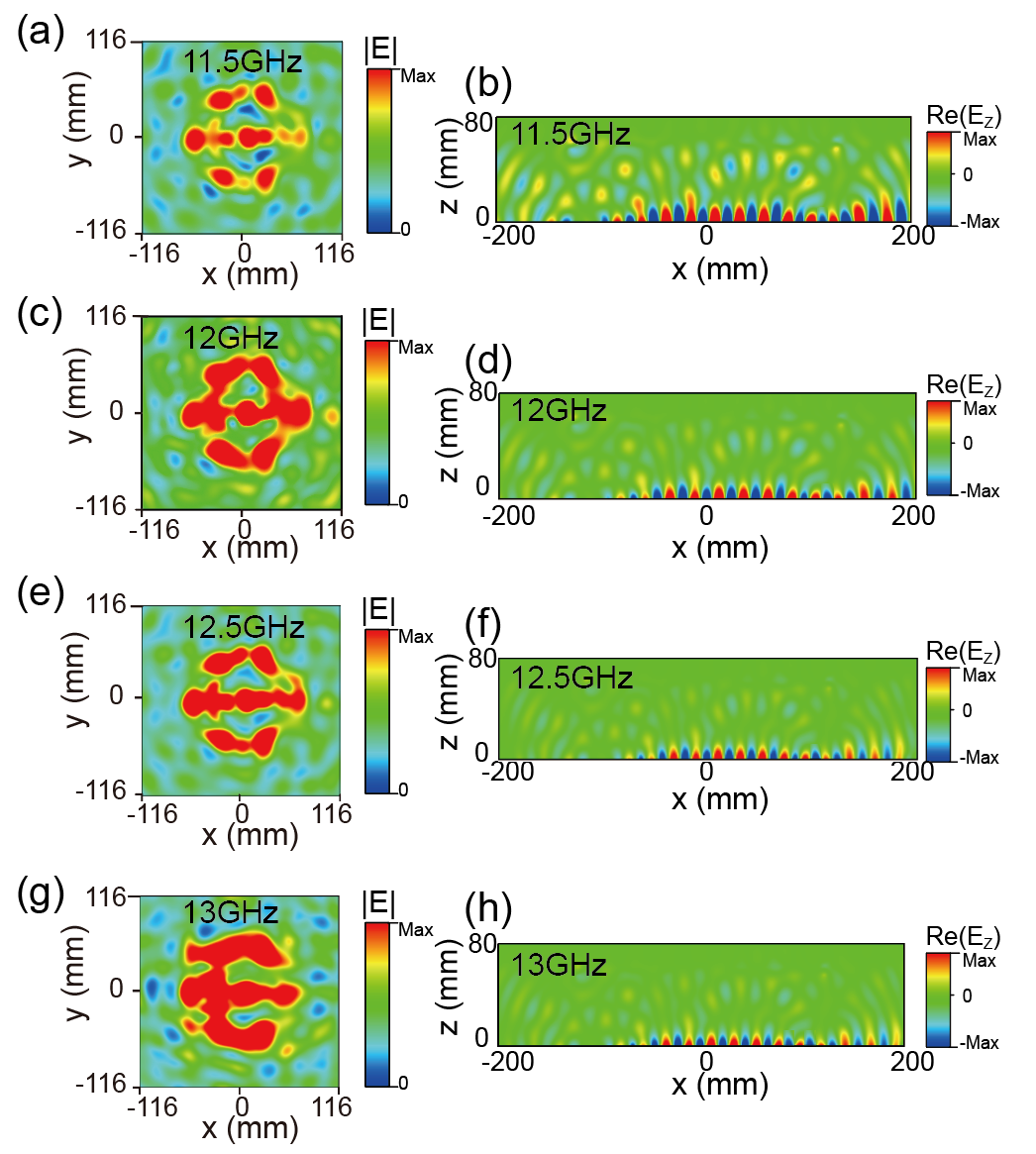


Figure S8. Simulated (a,c,e,g) electric-field patterns in the x-y plane at *z* = 125 mm of cross-polarized mode and (b,d,f,h) near-field Re[*E_z_*] patterns of the co-polarized mode, generated by meta-device II under LCP wave illumination from 11.5 to 13 GHz. It is noted that the working band of such bifunctional metasurface is about 1.5 GHz (11.5- 13 GHz).

# G. Structural details of three bi-functional metadevices in Fig. 5


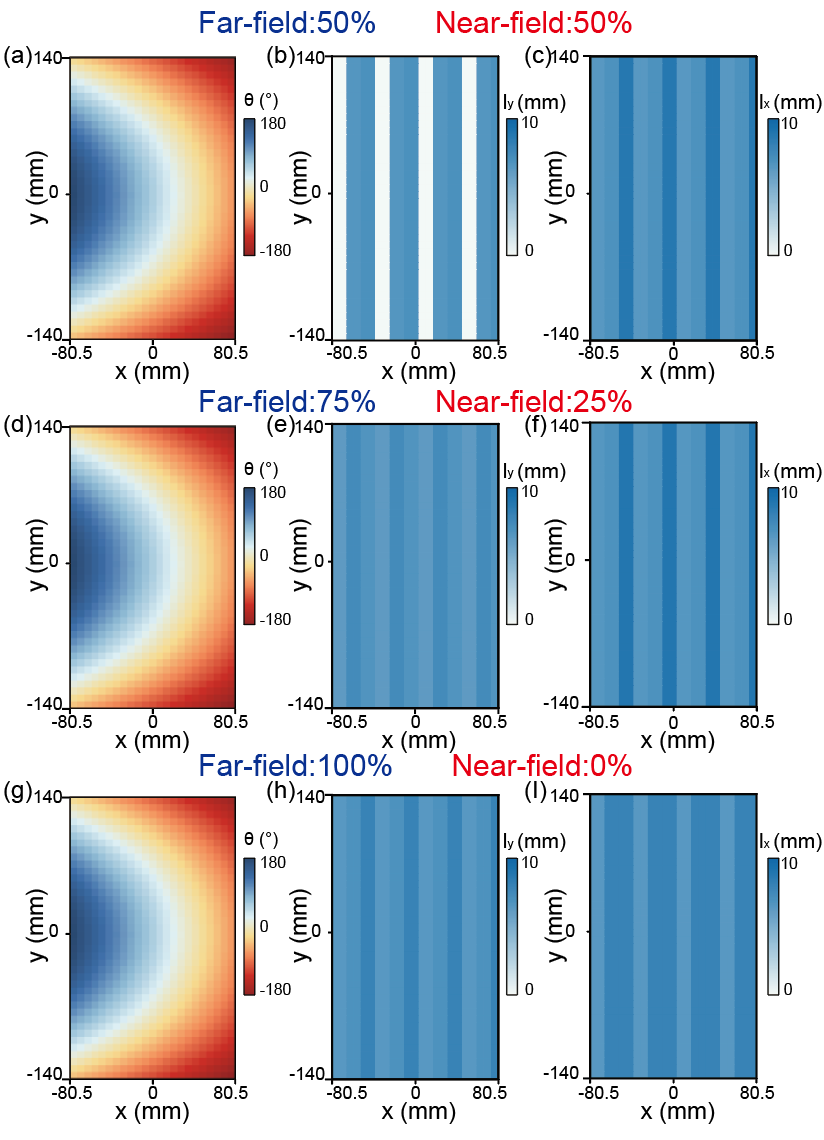


Figure S9. Distributions of (a, d, g) $\theta(x,y)$, (b, e, h) $l_{y}(x,y)$ and (c, f, i) $l_{x}(x,y)$ for the three bi-functional meta-devices shown in Fig. 5. They can generate both far-field and near-field wavefronts with different energy distributions.

# References:

[1] Huang L, Chen X, Bai B, et al. Helicity dependent directional surface plasmon polariton excitation using a metasurface with interfacial phase discontinuity[J], Light Science & Applications, 2013, 2(3):e70.

[2] Luo W, Xiao S, He Q, et al. Photonic Spin Hall Effect with Nearly 100% Efficiency[J]. Advanced Optical Materials, 2015,3(8):1102-1108.
